# Female Sexual Dysfunction in Medical Residents: Relationships with sociodemographic and clinical Factors

**DOI:** 10.1192/j.eurpsy.2025.2287

**Published:** 2025-08-26

**Authors:** R. Masmoudi, S. Ajmi, M. Mnif, M. Bouhamed, I. Feki, R. Sellami, J. Masmoudi

**Affiliations:** 1 Psychiatry A department, Hedi Chaker hospital university, Sfax, Tunisia

## Abstract

**Introduction:**

Female sexual dysfunction (SD)has gained attention in recent years. Multiple factors contribute to women’s sexual vulnerability, including neurobiological, sociocultural, psychological, and interpersonal factors.

**Objectives:**

To assess the prevalence of SD and to determine sociodemographic and clinical factors related to this dysfunction.

**Methods:**

We conducted a cross-sectional and descriptive study including married and sexually active female medical residents from different specialties.Data were collected using a self-questionnaire published by GOOGLE FORMS. The questionnaire included sociodemographic characteristics, substance use, marital life information, professional data, and information related to sexual life. Female Sexual Function Index (FSFI) was used to assess female sexual dysfunction.

**Results:**

A total of 65 medical residents completed the online questionnaire. Four residents (6.20%) considered the work environment to be unfavorable, 3.1% were smokers and 55.4% had children. The average frequency of sexual intercourse was 6.61 ± 4.18 times per month. Nine participants (13.8%) reported having sexual intercourse under partner pressure. Sexual dysfunction was observed in 49.2% of the cases. The factors correlated with the total FSFI score were an unfavorable work environment (p=0.02), smoking (p=0.007) and high frequency of sexual activities (p=0.02; r=0.28).The factors correlated with the “Desire” dimension were: sexual intercourse under partner pressure (p=0.002) and high frequency of sexual activities (p=0.001; r=0.41).The factors correlated with the “Arousal” dimension were: an unfavorable work environment (p=0.01) and psychiatric history (p=0.05).The “Lubrication” domain was associated with an unfavorable work environment (p=0.006) and smoking (p=0.02). The “Satisfaction” domain was associated smoking (p=0.004), sexual intercourse under partner pressure (p=0.03) and high frequency of sexual activities (p=0.003; r=0.36). The “Orgasm” domain was inversely correlated with age (p=0.04; r=-2.47), and it was associated with a high frequency of sexual activities (p=0.04; r=0.24). The “Pain” domain was correlated with the presence of children (p=0.02).

**Conclusions:**

The findings underscore the impact of various sociodemographic and clinical factors on sexual health and well-being. Addressing these underlying factors is essential for improving their sexual health and overall quality of life.

**Disclosure of Interest:**

None Declared

